# Structure and leaching characteristics of basaltic glass-ceramics for immobilizing simulated actinides La, Ce and Nd

**DOI:** 10.1371/journal.pone.0358195

**Published:** 2026-09-16

**Authors:** Qin Tong, Jun Liang

**Affiliations:** College of Intelligent Manufacturing, Mianyang Teachers’ College, Mianyang, China; VIT University, INDIA

## Abstract

Basaltic glass‑ceramics were prepared by melting basaltic glass, cooling the melt to the nucleation temperature, followed by a two‑stage nucleation‑crystallization heat‑treatment. Ce, Nd, and La were used to simulate U, Np, and Am in high‑level nuclear waste (HLW), respectively. The structure and leaching characteristics of basaltic glass-ceramics with different contents of simulated Actinides were investigated. The XRD and SEM results indicated the simulated actinides loading reaches 40 wt.%. When the content of simulated actinides exceeded 20 wt.%, the main crystal phase of the basaltic glass-ceramics was oxyapatite. As the content of the simulated actinides increases, the content of oxyapatite crystals increases, which indicates that an increase in the simulated nuclides content actually promotes the formation of basaltic glass-ceramic. The Raman results showed when the amount of simulated actinides exceeds 20 wt.%, the characteristic spectra of oxyapatite crystals appeared in the Raman spectra. The PCT test results showed the normalized mass loss of Na is the greatest, and the normalized leaching rates of rare earth elements are 4 orders of magnitude lower than that of Si and Na.

## Introduction

For the immobilization of high-level nuclear wastes (HLW), many ceramic phases are known to have better chemical durability than glass under typical storage conditions. However, the technologies associated with crystal ceramic manufacturing are generally more complex than those associated with glass production, especially in nuclear environments where remote processing facilities are required [1,2]. Glass-ceramics are promising nuclear waste forms with excellent stability and facile fabrication processes. Existing research indicates that apatite can more effectively accumulate Ln^3+^ than zirconolite [3,4]. Although oxyapatite Ca_2_Nd_8_(SiO_4_)_6_O_2_ exhibits slightly inferior radiation resistance, it only undergoes minor amorphization under self-irradiation and maintains relatively favorable chemical stability [5,6]. Ge et al. synthesized garnet-derived oxyapatite glass-ceramics at low temperature for Nd-simulated trivalent actinide immobilization. Rising Nd_2_O_3_ promotes oxyapatite formation, with maximum 60 wt.% Nd loading. Around 70%–80% Nd is locked in oxyapatite via crystal-glass dual barrier, and the material presents low Nd leaching rate of ~10^-5^ g m^-2^ d^-1^ after long-term leaching [7]. Yu et al. prepared Nd/Ce co-doped oxyapatite borosilicate glass-ceramics via melting-crystallization to immobilize simulated trivalent and tetravalent actinides. Nd addition suppressed CeO_2_ precipitation and promoted stable oxyapatite formation, transforming partial [BO_3_] into [BO_4_] units at high Nd loading. PCT (the ASTM Product Consistency Test Method) leaching tests revealed extremely low Ce/Nd leach rates at 10^-7^ g m^-2^ d^-1^ after 28 days, verifying outstanding chemical durability for nuclear waste immobilization [8]. Due to matching oxidation states and similar ionic radii, the ionic radii of Ce^4+^, Ce^3+^, Np^4+^, Np^3+^, Nd^3+^, Am^3+^, La^3+^, and U^3+^ are 0.087 nm, 0.101 nm, 0.087 nm, 0.110 nm, 0.098 nm, 0.098 nm, 0.103 nm and 0.102 nm, respectively. In conventional experiments, La, Ce, and Nd are typically considered excellent surrogate nuclides for U, Np, and Am [9,10]. The research team of the present author conducted relevant experiments. Different mass fractions of La_2_O_3_, Nd_2_O_3_, CeO_2_ and simulated actinide elements were incorporated into basaltic glass (obtained from basalt through high-temperature melting and cooling), followed by melting treatment at 1400 °C. La_2_O_3_ solubility in basaltic glass reaches 46 wt.%, and apatite precipitates above 56 wt.%. A low content of La_2_O_3_ contributes to glass‑network repair, while a high content of La_2_O_3_ degrades the glass network. The La leaching rate is three orders lower than for matrix ions [11]. Basaltic glass dissolves maximum 30 wt.% Nd_2_O_3,_ overloading forms oxyapatite. Moderate Nd_2_O_3_ improves thermal stability, and the Nd leaching rate is 4–5 orders lower than other elements after PCT test [12]. Co-doped basaltic glass holds simulated actinides up to 36 wt.%. Excessive doping precipitates oxyapatite Ca_2_Nd_8_(SiO_4_)_6_O_2_. Partial Si-O bonds break, while rare-earth leaching is four orders lower than host constituents [13]. Ce-doped basaltic glasses were prepared. The waste loading of CeO_2_ in basaltic glass reached~18 wt.%, and CeO_2_ crystals precipitated when the CeO_2_ content reached 20 wt.%. Roughly half Ce^4+^ converts to Ce^3+^ during melting. The glass exhibits higher thermal stability and lower Ce leaching rate with increasing CeO_2_ content [14]. The loading of simulated waste (prepared with reference to a Chinese power reactor) in basaltic glass reached~25 wt.%, and NiFe_2_O_4_ crystals precipitated when the simulated waste content reached 25 wt.%. When the content of simulated waste reached 40 wt.%, a small amount of yellow material precipitated from the upper surface of the sample [15]. Prepared samples possess favorable thermal stability and low mass loss per MCC-1 leaching tests [15]. Previous studies confirmed that basaltic glass possesses high loading capacity for Ce, Nd, La actinide surrogates and excellent aqueous durability with ultra-low nuclide leaching rates [11–15]. Accordingly, this work further investigates the existing forms and leaching mechanism of simulated radionuclides in basaltic glass-ceramics (prepared by heat-treatment of basalt glass).

## Experimental methods

### Fabrication of simulated waste glass

Nd, Ce and La were used to simulate U, Np and Am in nuclear waste. The contents of simulated nuclide oxides (Nd_2_O_3_, CeO_2_ and La_2_O_3_) were calculated based on the proportions of various nuclides in the original data (Table 1) of a power reactor in China, and the calculated results are presented in Table 2. The basaltic glass (Sichuan Aerospace Tuoxin Basalt Industrial Co., Ltd., China) which components were listed in Table 3 was mixed with the simulated actinides.

**Table 1:** The concentration of main components in High Level Liquid Waste (HLLW) of a power reactor in China [15].

Components	Concentration (g·L^-1^)	Components	Concentration (g·L^-1^)	Components	Concentration (g·L^-1^)
Alkali metal nitrates	26	Fe(NO_3_)_3_	1	Actinide nitrates	3
Alkali earth metal nitrates	10	Zr(NO_3_)_4_	23	Hypoactinide nitrates	3
Cr(NO_3_)_3_	2	Mo(NO_3_)_6_	30	others	24
Al(NO_3_)_3_	1	Rare earth nitrates	37	total	160

**Table 2:** Proportion of each oxide in the mixture of Nd_2_O_3_, CeO_2_ and La_2_O_3_ (wt.%).

oxide	Nd_2_O_3_	CeO_2_	La_2_O_3_
mass ratio	30.82	20.44	48.74

**Table 3:** Components of basaltic glass from the manufacturer.

Components	SiO_2_	Al_2_O_3_	Fe_2_O_3_ total	FeO total	CaO	MgO	TiO_2_	Na_2_O	K_2_O
Mass fraction(%)	51.30	15.54	6.52	4.15	10.12	5.80	1.60	3.40	0.73

A mixture of simulated actinides with different mass ratio (0, 10, 20, 30, 36 and 40 wt.%, respectively) was added to the basaltic glass, which was labelled as GCX (X = 0, 10, 20, 30, 36, 40). Table 4 lists the components of each sample. The raw materials were mixed in agate jars by planetary ball-milling (QM-3SP2, Nanjing NanDa Instrument Plant, Nanjing, China) for 30 min. Samples (approximately 30 g) of each mixture were heated in a corundum crucible in air at 1400 °C (10 °C/min ramp-up) for 3 h to form homogeneous melts. The homogeneous melt was cooled to the nucleation temperature of 800 °C at the identical rate, held for 2 h, then heated to the crystallization temperature of 980 °C with another 2 h holding, and finally furnace‑cooled to room temperature.

**Table 4:** Components of basaltic glass-ceramics (%).

Samples		basaltic glass	Nd_2_O_3_	CeO_2_	La_2_O_3_
GC0	wt./mol.	100	0	0	0
GC10	wt.	90	3.082	2.044	4.874
	mol.	89.93	2.56	3.32	4.19
GC20	wt.	80	6.164	4.088	9.748
	mol.	79.87	5.12	6.64	8.37
GC30	wt.	70	9.246	6.132	14.622
	mol.	69.85	7.68	9.95	12.54
GC36	wt.	64	11.095	7.358	17.546
	mol.	63.83	9.21	11.94	15.04
GC40	wt.	60	12.328	8.176	19.496
	mol.	59.81	10.22	13.26	16.71

### Characterization

Glass powder samples with particle sizes smaller than 80 μm were adopted for X-ray diffraction testing, which was conducted on an X’Pert PRO diffractometer with Cu Kα radiation (λ = 1.54187 Å). A Renishaw InVia laser Raman spectrometer equipped with a CCD detector was utilized to collect the Raman spectra of the prepared samples. The tests were performed at an excitation wavelength of 785 nm, and the scanning wavenumber range was set from 200 cm^-1^ to 1300 cm^-1^. A Carl Zeiss Ultra 55 field emission scanning electron microscope (FE-SEM) coupled with an energy dispersive X-ray spectrometer was employed to characterize the microscopic structure and morphological features of the specimens. The testing parameters were set at an accelerating voltage of 15 kV and a working distance of around 7.6 mm. For microscopic observation, flat block specimens acquired from the sample cross-sections were pretreated via etching with 10% hydrofluoric acid (HF) for 30 s and gold spraying. Subsequently, elemental scanning and spot analysis on the glass surface were carried out using energy dispersive X-ray spectroscopy (EDX).

### Static leaching experiment

The aqueous durability of the obtained samples was evaluated by the ASTM Product Consistency Test Method B (PCT-B) in static leaching experiments at 90 ± 2 °C [16]. The powder samples were sieved between 75 and 150 μm, cleaned with deionized water and dried. Three grams of dried powder from each sample was put into a sealed Teflon reactor filled with 15 mL of deionized water. The leachates were extracted on the 1st, 3rd, 7th, 14th and 28th days for determination. The concentrations of the main elements in the leaching solutions were obtained via inductively coupled plasma-mass spectrometry (ICP-MS, 7700X, Agilent Technologies, Santa Clara, CA, USA) [11–14]. The normalized mass loss *NL*_i_ (g m^-2^) can be expressed by Equation (1):


$$\begin{array}{c} NL_{\text{i}}=\frac{C_{\text{i}}}{f_{\text{i}}\left(\frac{S}{V}\right)} \end{array}$$ (1)

where *C*_i_ is the concentration of the i-th element in the leaching solution (g m^-3^), *f*_i_ is the mass fraction of the i-th element in the sample, *V* is the volume of the leaching solution (m^3^), and *S* is the surface area of the sample powder (m^2^). The surface area *S* is calculated via Equation (2) [12,14].


$$\begin{array}{c} S=\text{6}mass/\rho d \end{array}$$ (2)

where ρ is the density of glass in g cm^-3^, mass is the mass of leached powder in g, *d* is the particle size diameter in cm, and the average diameter of particles in the 100–200 mesh (75–150 μm) size fraction is 1.12 × 10^–4^ m. The density of glass is measured via the pycnometer method.

## Results and discussion

### Phases and microstructure

**Fig 1:**
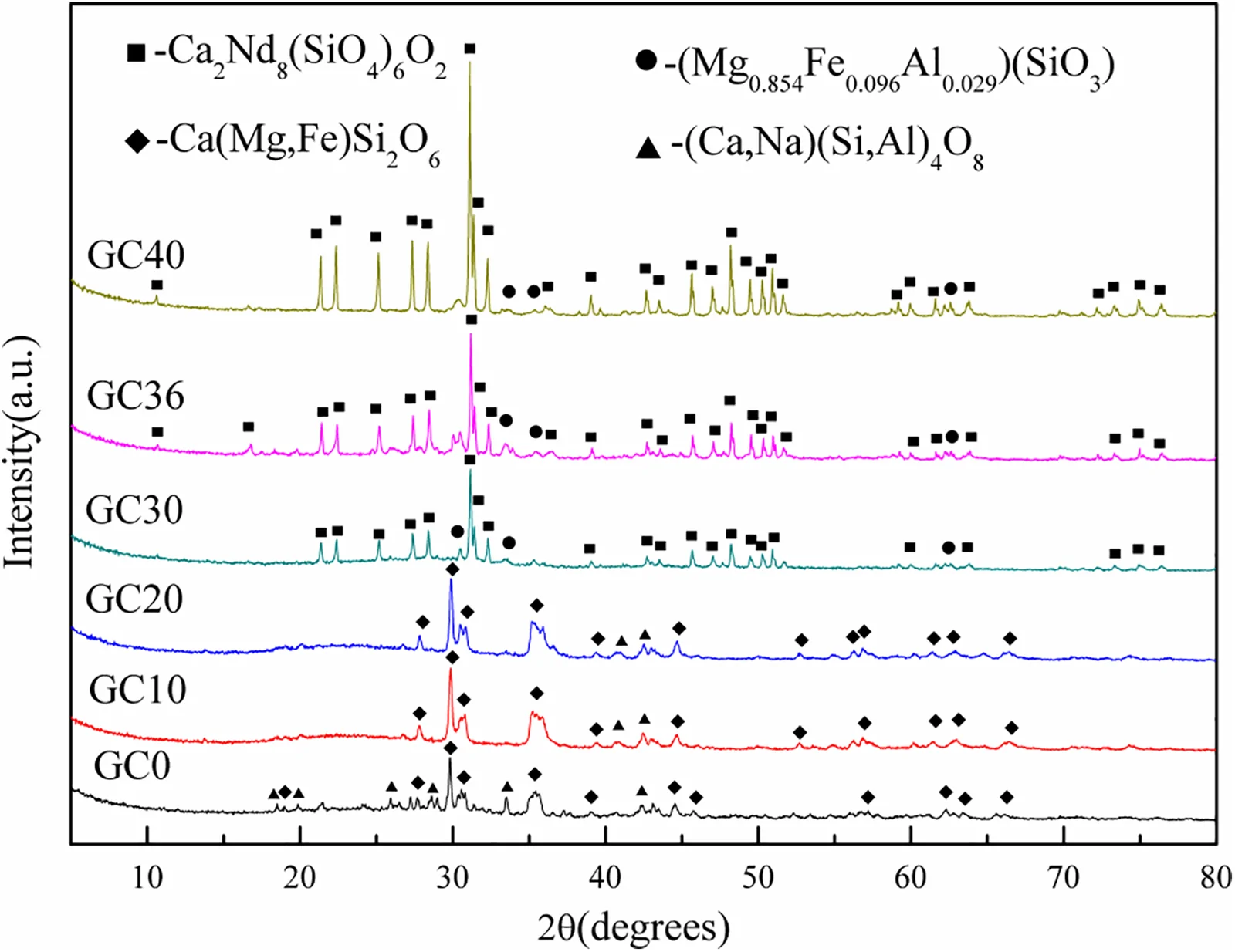
XRD patterns of GC0-GC40 samples.

The X-ray diffraction patterns of the GC0-GC40 samples (Fig 1) revealed that a large number of crystals were precipitated in these samples. When the content of the simulated actinides was less than 20 wt.%, the crystals precipitated in the samples were mainly diopside (Ca(Mg,Fe)Si_2_O_6_, PDF#72–1379) and plagioclase ((Ca,Na)(Si,Al)_4_O_8_, PDF#18–1202). When the content of the simulated actinides was 20 wt.%, a small amount of the target crystalline phase oxyapatites were precipitated. When the content of the simulated actinides reached 40 wt.%, the crystals precipitated from the samples were mainly oxyapatite (Ca_2_Nd_8_(SiO_4_)_6_O_2,_ PDF#78–1038), with a small amount of enstatite ((Mg_0.854_Fe_0.096_Al_0.029_)(SiO_3_), PDF#76–2427). This result demonstrates that the maximum waste loading of basaltic glass-ceramic reaches 40 wt.%. Compared with the conclusions of our previous studies, the loading capacity when using basaltic glass-ceramic to immobilize simulated waste is higher than that of basaltic glass, which only accommodates up to 36 wt.% simulated actinides [13].

The diffraction peaks of oxyapatite slightly shift toward lower angles relative to standard PDF data. This phenomenon is closely associated with the doping of Ce and La elements. The doped Ce^3+^ and La^3+^ can partially substitute for Ca^2+^ in the crystal lattice. The ionic radii of Ce^3+^, La^3+^ and Ca^2+^ are 0.101 nm, 0.103 nm and 0.1 nm, respectively. The differences in ionic radius result in variations of the lattice parameters [17–19].

### SEM and EDX analysis

**Fig 2:**
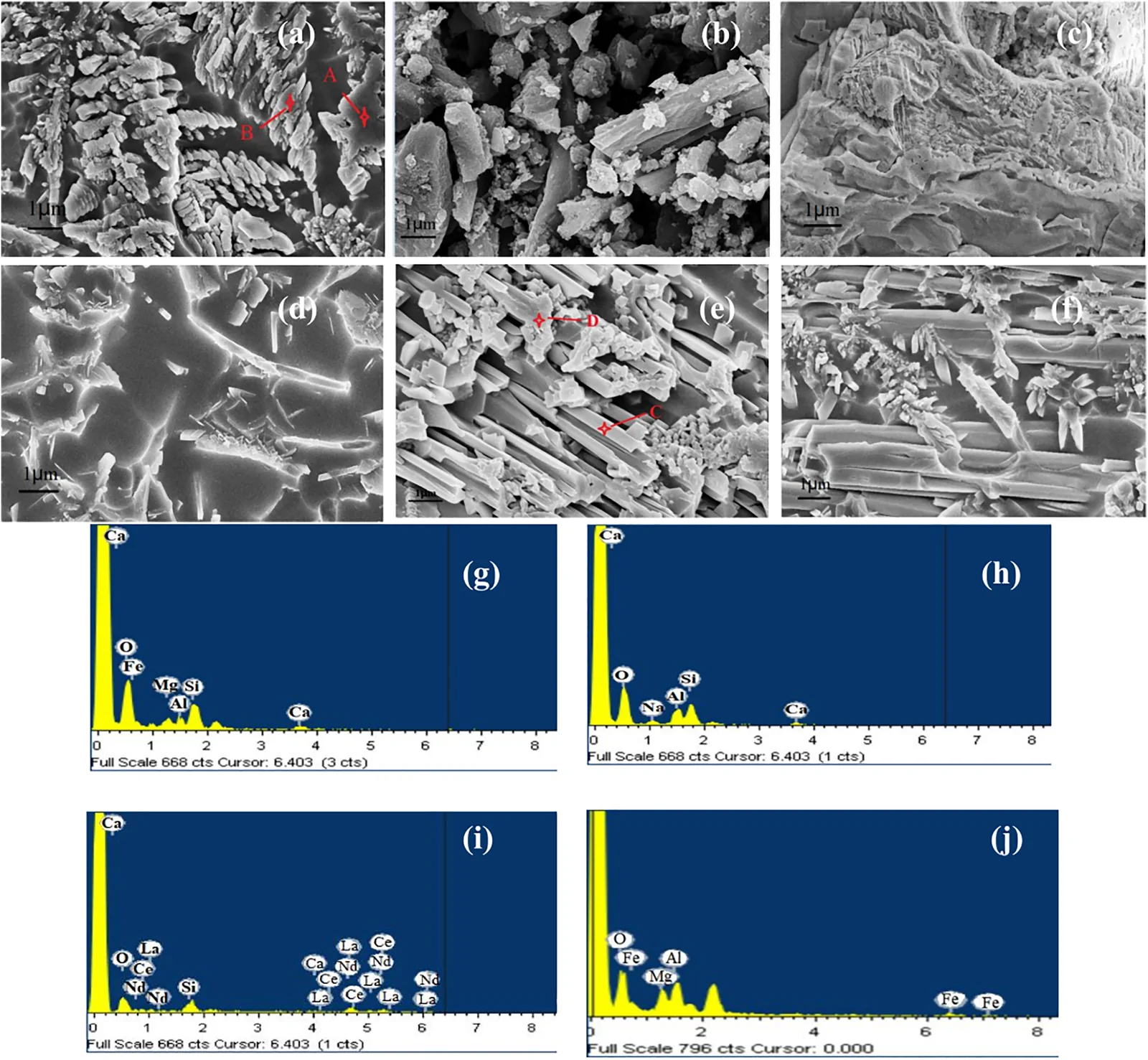
(a)-(f) SEM image of GC0-GC40; (g)-(j) EDX spectra of A, B, C and D in (a) and (e) image region.

The SEM images and EDX spectra of GC0-GC40 samples obtained by scanning electron microscopy and energy spectrometer were shown in Fig 2. As shown in Fig 2(a)–2(f), massive crystals (marked A) and clustered crystals (marked B) dominated the microstructures of GC0, GC10 and GC20, while slender rod‑like crystals (C) and granular grains (D) were the major crystalline phases in GC30, GC36 and GC40. Fig 2(g)–2(j) show the elemental compositions of crystals with different morphologies. Massive crystals were mainly composed of elements Si, Ca, Mg, Fe, Al and O. Cluster crystals were mainly composed of elements Si, Ca, Na, Al and O. Slender rod-shaped crystals were mainly composed of elements Si, Ca, Nd, Ce, La and O. Granular crystals were mainly composed of elements Mg, Fe, Al, Si and O.

According to the EDX spectra of crystals and XRD results, it can be inferred that the massive crystals were diopside, the cluster crystals were plagioclase, the slender rod-shaped crystals were oxyapatite, and the granular crystals were enstatite. And as the content of the simulated actinides increased, it significantly promoted the growth of the oxyapatite crystals, which is consistent with the research conclusions of Ge et al. and Yu et al [7,8]. This indicates that an increase in the content of simulated nuclides actually promotes the formation of basaltic glass‑ceramic with oxyapatite as the main crystalline phase.

### Raman analysis

**Fig 3:**
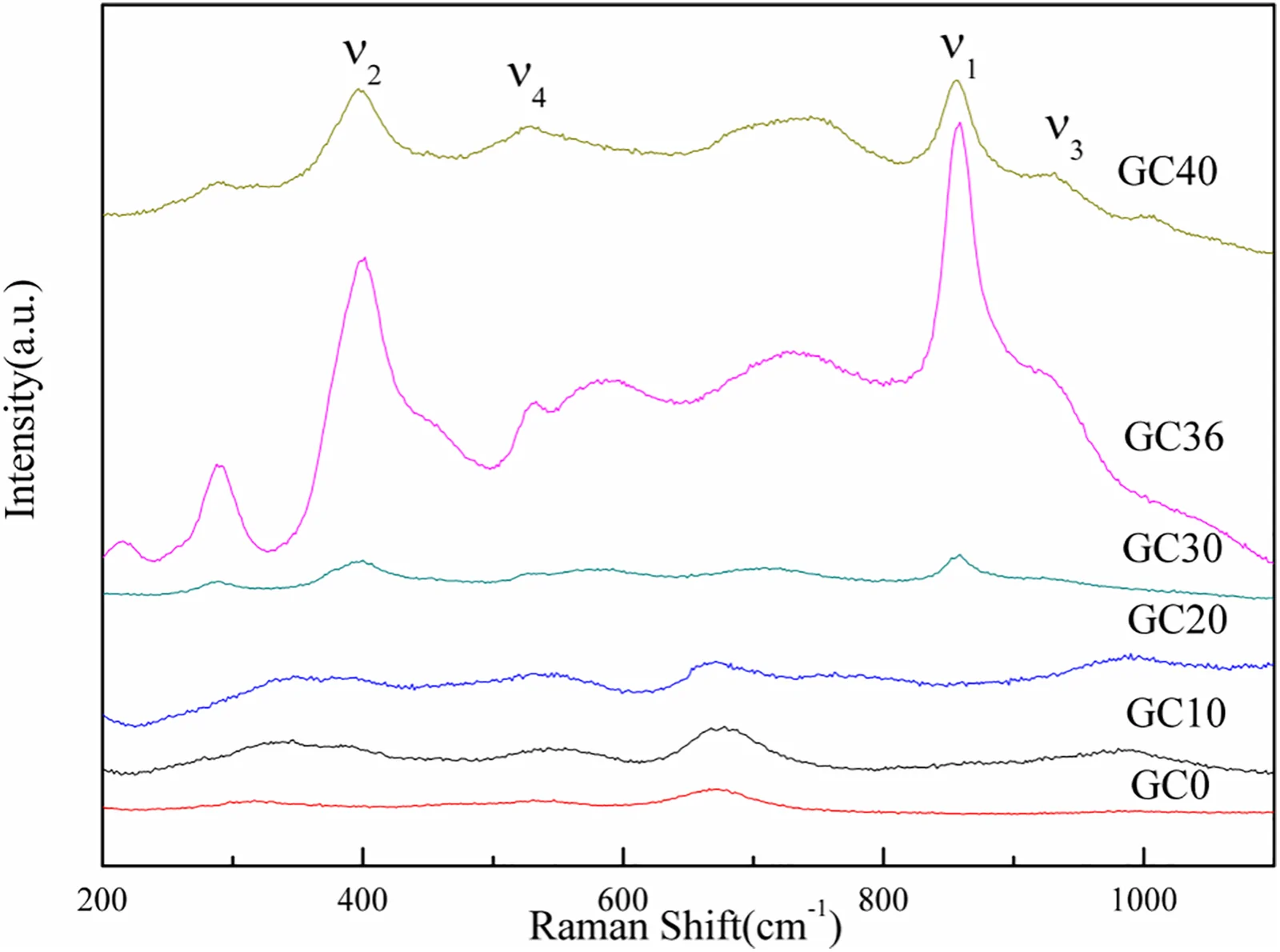
Raman spectra of GC0-GC40.

Fig 3 shows the Raman spectra of samples GC0-GC40 with a content of 0–40 wt.% in the range of 200 cm^-1^ to 1200 cm^-1^. These curves indicated that when the content of simulated actinides exceeded 20 wt.% (GC30-GC40), their Raman spectra were highly consistent with those reported in the literature for oxyapatite‑type structures [20-22]. Two prominent scattering peaks appear at the band of 390 cm^-1^ and 850 cm^-1^. The band of 390 cm^-1^ is assigned to the symmetric bending mode ν_2_ of SiO_4_ tetrahedra, while the peak near the band of 525 cm^-1^ originates from the antisymmetric bending mode ν_4_. In addition, the scattering peak around the band of 850 cm^-1^ corresponds to the symmetric stretching mode ν_1_ of SiO_4_ tetrahedra, and the weak peak near the band of 920 cm^-1^ is attributed to the antisymmetric stretching mode ν_3_. By contrast, the scattering peaks below 350 cm^-1^ originate from other external modes, such as translational and rotational oscillations of SiO_4_ tetrahedral units [20–22]. However, when the amount of simulated actinides was less than 20 wt.% (GC0 and GC10), the Raman spectra of the samples were significantly different from those of GC30-GC40 samples. Among them, the band near 1000 cm^-1^ was mainly attributed to the symmetric bending and stretching vibration of Si-O_n_b (non-bridging oxygen), the band of 670 cm^-1^ was mainly related to the symmetric bending vibration of Si-O-Si, and the band of 300–400 cm^-1^ belonged to the asymmetric bending vibration of Si-O-Si [23–27]. The above bands mainly belonged to the characteristic spectra of diopside, and the bands around 500–600 cm^-1^ were mainly related to the O-Si-O bending vibration in diopside (559 cm^-1^) and the oxy-bridge bending vibration in plagioclase (507 cm^-1^). Due to the small crystal content in the sample GC20, the Raman spectrogram of amorphous glass was mainly shown [28–30].

### Leaching characteristics

**Fig 4:**
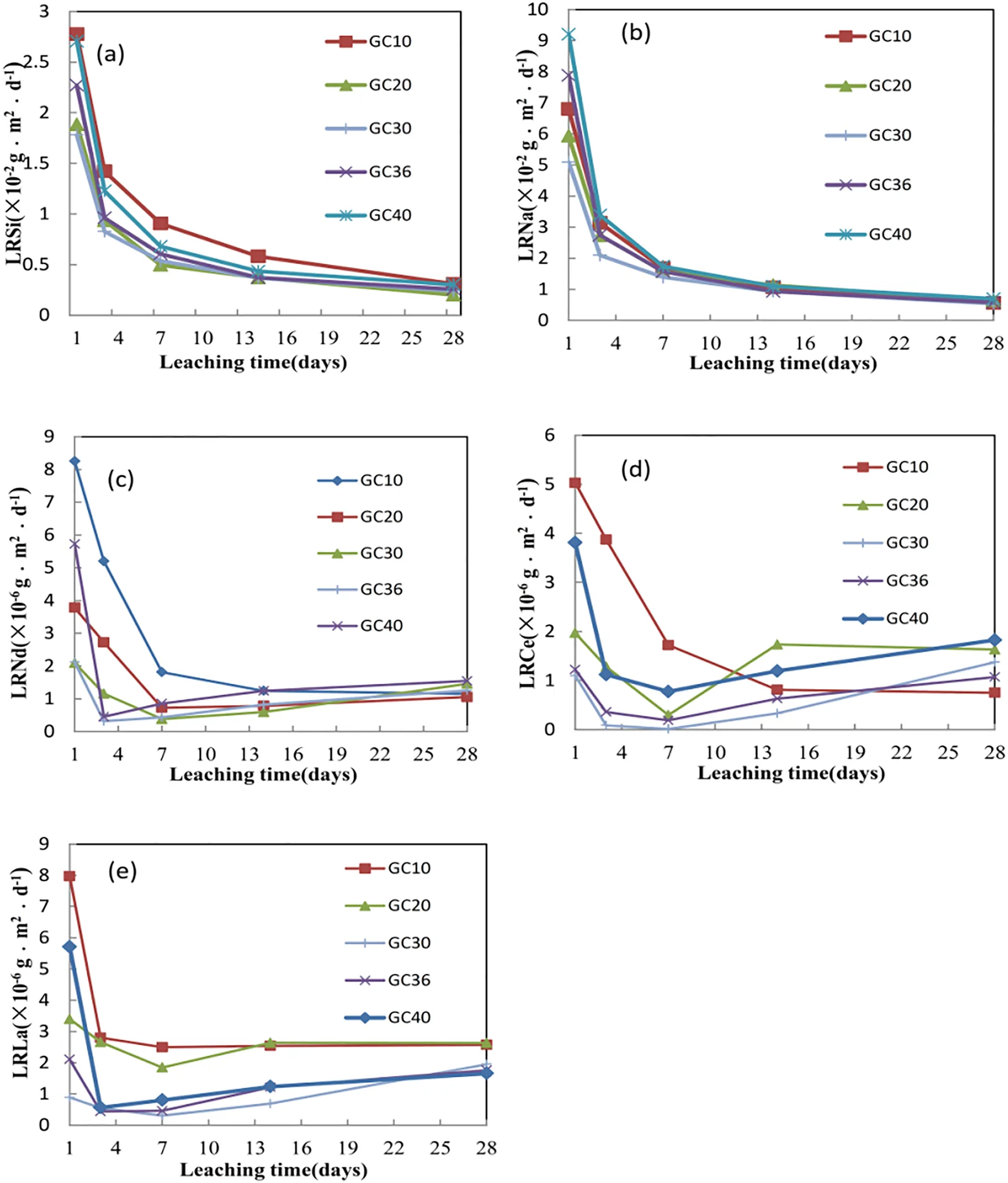
Normalized elemental leach rates of Si(a), Na(b), Nd(c), Ce(d) and La(e) of GC10-GC40.

The normalized mass losses of Si (*LR*_Si_), Na (*LR*_Na_), Nd (*LR*_Nd_), Ce (*LR*_Ce_) and La (*LR*_La_) after 28 days were depicted in Fig 4. All elements exhibited a sharp mass loss within the initial 7 days, followed by small‑magnitude variations in the later stage. This trend was attributed to the formation of a gel layer on the glass surface, which inhibits elemental diffusion [31,32]. From day 7 onwards, the normalized mass losses of Nd, Ce and La increased slightly, which may be due to the failure of the glass structure.

After 28 days immersion, the leaching rates of each element were as follows: *LR*_Si_ = 0.201 ~ 0.309 × 10^-2^ g m^-2^ d^-1^, *LR*_Na_ = 0.540 ~ 0.696 × 10^-2^ g m^-2^ d^-1^, *LR*_Nd_ = 1.053 ~ 1.545 × 10^-6^ g m^-2^ d^-1^, *LR*_Ce_ = 0.752 ~ 1.825 × 10^-6^ g m^-2^ d^-1^, *LRL*_a_ = 1.655 ~ 2.631 × 10^-6^ g m^-2^ d^-1^. Among them, the normalized mass loss of Na is the greatest. This is probably related to the occurrence state of Na in the glass network and its chemical reaction with water [33]. The order of magnitude of the normalized leaching rates for all elements is consistent with the previous research results of the author’s research team [11–15]. Moreover, the normalized leaching rate of rare earth elements is 4 orders of magnitude lower than that of Si and Na. In general, the leaching characteristics of basaltic glass-ceramics were very good, and the normalized mass losses of all elements meet the expectations of nuclear industry standards (<1 g m^−2^ d^−1^) [34].

## Conclusions

Basaltic glass‑ceramics with oxyapatite as the main crystalline phase were fabricated from a basaltic glass matrix using this melt‑cooling plus two‑stage nucleation‑crystallization heat‑treatment route. XRD and microstructure analysis revealed that as the content of the simulated actinides increases, the content of oxyapatite crystals increases, which indicated that an increase in the simulated nuclides content actually promotes the formation of basaltic glass-ceramic. When the content of simulated actinides exceeded 20 wt.%, the main crystal phase of the basaltic glass-ceramics was oxyapatite accompanied by a small amount of enstatite. Raman spectra showed when the amount of simulated actinides exceeds 20 wt.%, the characteristic spectra of oxyapatite crystals appeared in the Raman spectra. The PCT test results showed the normalized mass loss of Na is the greatest, and the normalized leaching rate of rare earth elements is 4 orders of magnitude lower than that of Si and Na.
